# Personal Health Budget: a new rehabilitation approach for severe mental illness within a caring community

**DOI:** 10.1192/j.eurpsy.2022.635

**Published:** 2022-09-01

**Authors:** L. Pelizza, D. Maestri, E. Leuci, G. Paulillo, P. Ceroni, E. Quattrone, P. Pellegrini

**Affiliations:** AUSL di Parma, Department Of Mental Health, Parma, Italy

**Keywords:** Personal Health Budget, rehabilitation, Community, mental health care

## Abstract

**Introduction:**

Personal Health Budget (PHB) has been provided to consumers with severe mental illness within a policy shift toward a person-tailored mental healthcare treatment based on individual unmet needs. PHB is an amount of money to support patient’s health and wellbeing needs, which is planned and agreed between patients and their local NHS team. It is not new money, but it may mean spending money differently so that patients can get the care that they need. However, evidence of beneficial effects of PHB is still scarce.

**Objectives:**

The aim of this study was to provide preliminary data on clinical and social benefits of adding PHB to a standard pharmacotherapy in patients with severe mental illness across a 24-month follow-up period.

**Methods:**

137 individuals with severe mental illness (aged 18–50 years) were recruited in one of the adult mental health services of an Italian Department of Mental Health. They completed the Global Assessment of Functioning scale, the Health of the Nation Outcome Scale and the Brief Psychiatric Rating Scale. Friedman’s test for repeated measure was used to assess the longitudinal stability of functioning and clinical parameters. A linear regression analysis was also performed.

**Results:**

A significant decrease in all GAF scale, HoNOS and BPRS scores along the 24 months of follow-up was observed. Regression analysis results specifically showed a relevant association between a PHB multiaxial intervention and the longitudinal reduction in BPRS ‘Negative Symptoms’ and HoNOS ‘Social Problems’ subscores.

**Conclusions:**

Our findings support the useful implementation of a PHB approach for severe mental illness patients within the Italian mental health service network.

**Disclosure:**

No significant relationships.

